# P-1942. Unexpected Cardiac Deaths in Patients with COVID-19 Infection

**DOI:** 10.1093/ofid/ofae631.2101

**Published:** 2025-01-29

**Authors:** Aimon Malik, Atika Jabeen, Hiam Chemaitelly, Anil G Thomas, Sherin Shams, Samah Saleem, Zain A Bhutta, Aftab M Azad, Abdullatif Al Khal, Laith J Abu-Raddad, Abdul-Badi Abou-Samra, Adeel A Butt

**Affiliations:** Hamad Medical corporation, Doha, Ad Dawhah, Qatar; Department of Emergency Medicine, Hamad Medical Corporation, Doha, Qatar, Doha, Ad Dawhah, Qatar; Weill Cornell Medicine - Qatar, Doha, Ad Dawhah, Qatar; Hamad Medical corporation, Doha, Ad Dawhah, Qatar; Hamad Medical Corporation, Doha, Ad Dawhah, Qatar; Hamad Medical corporation, Doha, Ad Dawhah, Qatar; Department of Emergency Medicine, Hamad Medical Corporation, Doha, Qatar, Doha, Ad Dawhah, Qatar; Department of Emergency Medicine, Hamad Medical Corporation, Doha, Qatar, Doha, Ad Dawhah, Qatar; Hamad Medical Corporation, Doha, Ad Dawhah, Qatar; Weill Cornell Medicine, Doha, Ad Dawhah, Qatar; Hamad Medical corporation, Doha, Ad Dawhah, Qatar; Weill Cornell Medicine, Doha, Ad Dawhah, Qatar

## Abstract

**Background:**

There are contradictory reports regarding the association of COVID-19 with sudden cardiac deaths. It is unclear whether these deaths are the result of known cardiac disease and risk factors or are unexpected based on prevalent risk factors. Our aim was to determine the association between COVID-19 infection and UCD among individuals who died of a cardiac cause in Qatar.

Study Flowsheet

**Figure:**
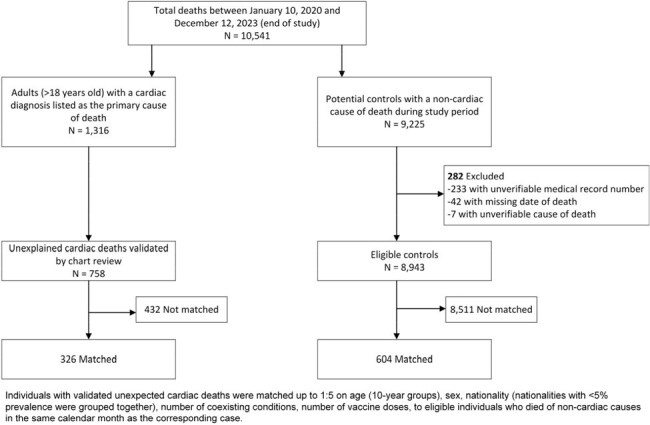

**Methods:**

All deaths in Qatar in adults >18 years old with any cardiac cause listed as the primary or immediate cause of death were individually reviewed to determine whether the death was unexpected based on the individual’s clinical risk profile. For each case of validated UCD, up to 5 controls with a non-cardiac primary cause of death were identified, matched on age groups, sex, nationality, number of coexisting conditions, and vaccine doses to individuals who died of non-cardiac causes in the same calendar month. Adjusted odds ratios (aOR) comparing odds of prior COVID-19 infection in cases relative to controls were estimated using conditional logistic regression.

Association between prior COVID-19 infection and incidence of Unexpected Cardiac Deaths (UCD) in the matched cohorts.

**Figure:**
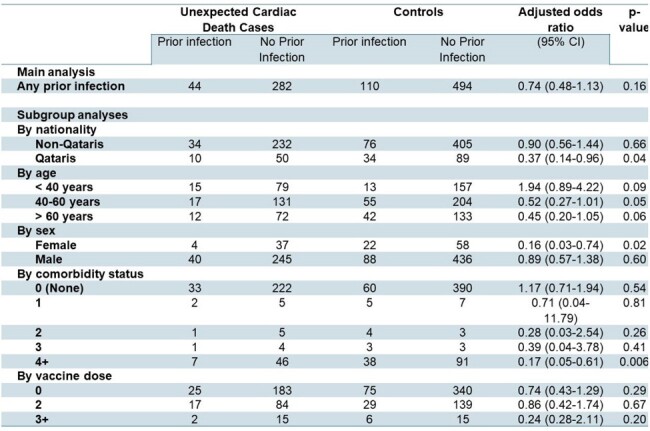

**Results:**

Among a total of 10,541 deaths, we matched 326 UCD cases to 604 controls with a non-cardiac cause of death. The median age was 48 years for cases and 49 years for controls, 87% were male, and 18% of cases and 20% of controls were Qatari nationals. Approximately three-quarters in each group had no documented comorbidities, and approximately two-thirds were unvaccinated. Prior COVID-19 infection was not associated with a higher risk of UCD (aOR 0.74, 95% CI 0.48, 1.13). No association was observed between prior COVID-19 and UCD in subgroup analyses by age groups, or vaccination status. Qatari nationals, females, and those with > 4 comorbidities were less likely to experience UCD associated with prior COVID-19.

**Conclusion:**

COVID-19 is not associated with an increased risk of UCD. The observed cardiac deaths are more likely due to the underlying risk profile of the decedents.

**Disclosures:**

Adeel A. Butt, MD, MS, Gilead Sciences: Grant/Research Support

